# The Spiritual Side of Nursing

**Published:** 1920-04-17

**Authors:** 


### THE SPIRITUAL SIDE OF NURSING.

"Have you considered the spiritual side of Nursing?" So writes a friendly critic, who thinks that I harp too constantly on the economic string. From the context I gather that my correspondent cherishes the consoling thought that the underpaid and overworked nurse enjoys, in addition to her worldly emoluments, a certain spiritual revenue which goes some distance to recompense her for any grievances that may happen to fall to her lot.

I wonder what my correspondent would think if, in answer to her question, I were to inquire: Have you considered the spiritual side of fighting? I hope that none has forgotten the tragic day in the summer of 1914, when the tocsin of war sounded and resounded from end to end of a world of peace and plenty. The best boys in the Empire responded to that call, and I can testify for many of them that before they took their departure they knelt at the Communion rail, feeling in their hearts' depths that they would come back never more. Parenthetically, I may mention that wherever these heroes went the nurse also went.

It may sound like a heresy, but I have no doubt in my mind that it is true to say that nursing has no more a spiritual side, and no less, than addressing envelopes. To the spiritually-minded all work is sacred. The word "secular" was coined for and by the secularist. I am glad that this question has been frankly put. I have for long been conscious that a large number of people who dwell on the "vocation" question take into serious account the spiritual wages which a devoted and conscientious nurse derives from her employment. I should be sorry to charge these people with being dishonest. On the contrary, they are, in the main, not alone honest, but high-minded. What is wrong is that they do not think along logical lines, and, failing to do so, their sense of justice is biased. I have witnessed abundant instances of this during the war—lavish remuneration to one class of workers and absolute parsimony to conscientious and devoted, albeit poor, volunteers. The *arrière pensée* is that there is a spiritual side, which means spiritual remuneration.

The argument is mean and contemptible, however well meant. It amounts to this, that you put the nurse on a pedestal, praise her, and halo her, with the deliberate object and intention of getting her to work "on the cheap." "Render unto Cæsar the things that are Cæsar's." I have no desire to get behind this commandment; it needs neither addition nor qualification; it is a perfect prescription for the entire economic disorder. But we allow ourselves to be tricked into false doctrines and erroneous methods of thinking by the misuse of the King's English. The most noxious expression of recent times was "war charities," an expression which found its way into Acts of Parliament. Had this been "war dues" or "war tribute" the effect on the public mind would have been wholly different. The nation's civilian effort was no charity. It was an imperative obligation, a debt of honour, and although many people have decried the Nation's Tribute to Nurses and the *Daily Telegraph* Shilling Fund appeals as derogatory to nurses, by making them the objects of "charity," the same principle applies. The nurse is toiling strenuously for the nation, and her wages should be paid, not alone willingly but generously.

It appears to me that the nurse and her duties have been for years, if not always, surrounded by clouds of camouflage, and, furthermore, that this false show has extended to the voluntary hospital system, a fact which accounts in a large degree for the present financial plight of these institutions. The citizen has not been taught his duty. He also thinks that the nation's sick should be healed at the expense of people who derive spiritual consolation from being philanthropic. And he selfishly settles down to letting others bear his burden.

From everybody's standpoint, except her own, the nurse's problem is purely economic. She performs necessary and specific duties which demand a high-class training as well as certain moral and physical attributes. Into the spiritual realm it is an impertinence to intrude. The scullion of a King may live and move on a higher spiritual plane than his royal master. But this does not imply that either of them is underpaid or overpaid.

It is most damaging to the nursing profession and to the nursing efficiency of the country that the public are not educated to insist on economic reform. The only reasonable employers of the nurse are the War Office and the Admiralty. There may be an absence of sentiment, but the hours are satisfactory, the pay progressive, and a pension is assured. Until civil nursing is graded up to this standard the best candidates for the profession will turn aside and seek employment elsewhere.

